# *Notes from the Field:* Botulism Type B After Intravenous Methamphetamine Use — New Jersey, 2020

**DOI:** 10.15585/mmwr.mm6939a4

**Published:** 2020-10-02

**Authors:** Michelle A. Waltenburg, Valerie A. Larson, Elinor H. Naor, Timothy G. Webster, Janet Dykes, Victoria Foltz, Seth Edmunds, Deepam Thomas, Joseph Kim, Leslie Edwards

**Affiliations:** ^1^Epidemic Intelligence Service, CDC; ^2^Division of Foodborne, Waterborne, and Environmental Diseases, National Center for Emerging and Zoonotic Infectious Diseases, CDC; ^3^Morristown Medical Center, Morristown, New Jersey; ^4^Oak Ridge Institute for Science and Education, Oak Ridge, Tennessee; ^5^New Jersey Department of Health; ^6^ID CARE, Randolph, New Jersey.

On May 15, 2020, a White man aged 41 years arrived at an emergency department in New Jersey with a 2-day history of new onset blurred vision, double vision, ptosis, and difficulty swallowing. He was evaluated for cerebrovascular accident (CVA [stroke]), was found to have unremarkable computed tomography and magnetic resonance imaging brain scans, and was discharged with a diagnosis of diplopia (double vision). The following day, his symptoms worsened, and he visited a second emergency department with slurred speech, oral thrush, and facial weakness. Thorough skin and scalp examinations revealed peripheral phlebitis and sites of induration, but no abscesses or open wounds. He was admitted to the hospital with a diagnosis of CVA and treated with antifungal medications for oral and laryngeal candidiasis.

Past medical history was notable for methamphetamine use for approximately 20 years; the patient did not report any other illicit drug use. The patient reported he had only inhaled methamphetamine in the past; however, after a 2-week abstinence, he reported that he injected methamphetamine mixed with water intravenously approximately 24–48 hours before his symptoms began. The water came from a bottle that had been open in his home for an unknown duration. This history of recent intravenous drug use raised suspicion for botulism, a paralytic illness caused by botulinum neurotoxin (BoNT). To the patient’s knowledge, no one else who had injected the same batch of methamphetamine had had an adverse reaction.

Per New Jersey Reporting Regulations (NJAC 8:57),* the suspected illness was immediately reported to the New Jersey Department of Health. After consultation with CDC, heptavalent botulinum antitoxin was released by the CDC quarantine station in New York and administered to the patient within 24 hours of admission to the hospital. He did not require ventilatory support, and his symptoms of double vision, ptosis, difficulty swallowing, and facial weakness gradually improved until hospital discharge 5 days after antitoxin administration. The patient’s mild blurred vision persisted, and he was referred for vision rehabilitation, speech and language pathology, psychiatry, and infectious disease follow-up. Serum obtained before antitoxin administration tested positive for BoNT type B by the BoNT Endopep-MS assay, a mass spectrometry–based method that rapidly detects and differentiates active BoNTs, toxic substances that inhibit normal neuromuscular function (*1*).

Injection drug use is the leading cause of wound botulism in the United States; most cases occur in the western and southwestern United States,^†^ potentially associated with the supply and distribution of black tar heroin^§^ (*2*). Botulinum toxin type A is the most common toxin type among cases of wound botulism; in 2018, 47 of the 51 laboratory-confirmed cases of wound botulism were botulinum toxin type A, and injection drug use was reported by all wound botulism patients (Figure) (CDC, unpublished data, 2018). This case is notable for three reasons: 1) the rarity of botulinum toxin type B in wound botulism cases, 2) the occurrence in the northeastern United States, and 3) association with injection of methamphetamine rather than heroin.

**FIGURE:**
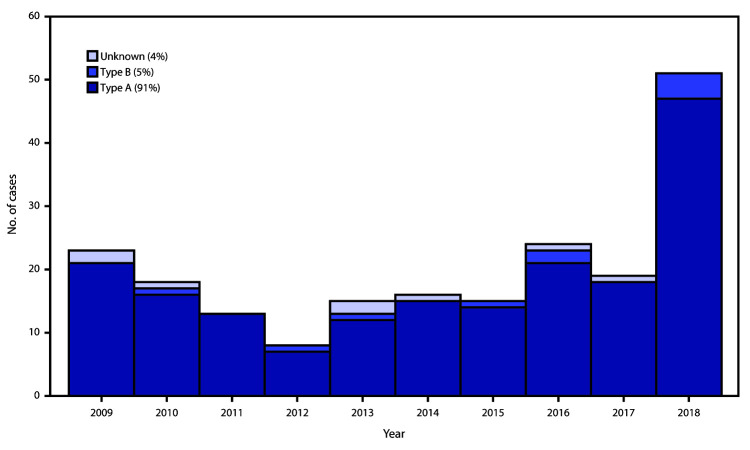
Laboratory-confirmed wound botulism cases, by year and botulinum toxin type — United States, 2009–2018* * 2018 data are provisional.

Although most wound botulism cases are caused by black tar heroin injection (*2*–*4*), this case highlights the need for awareness of the risks for and signs and symptoms of wound botulism^¶^ among all persons who inject drugs, as well as among clinicians caring for persons who inject drugs. Early recognition and treatment of botulism is critical to reducing morbidity and mortality, and broader awareness of risks and symptoms of wound botulism might prompt persons who have symptoms to seek medical care early and potentially facilitate an earlier diagnosis (*5*). Some signs of wound botulism (e.g., ptosis and altered phonation) might be interpreted as mental status changes associated with methamphetamine abuse, highlighting the importance of conducting a thorough neurologic examination to differentiate botulism from other diagnoses (*5*). This case further illustrates that mild wounds can harbor *Clostridia* bacteria that produce botulinum toxin (*5*); therefore, it is important for health care providers to consider wound botulism among patients with a history of injection drug use, even in the absence of a visible abscess or severe wound.

## Footnotes

*
https://www.state.nj.us/health/cd/reporting/acode/index.shtml .

†
https://www.cdc.gov/botulism/surv/2017/index.html .

§
https://www.dea.gov/sites/default/files/2018-07/DIR-001-17_2016_NDTA_Summary.pdf .

¶ https://www.cdc.gov/botulism/wound-botulism.html.

## References

[1] Barr JR, Moura H, Boyer AE, Botulinum neurotoxin detection and differentiation by mass spectrometry. Emerg Infect Dis 2005;11:1578–83. 10.3201/eid1110.041279 16318699 PMC3366733

[2] Werner SB, Passaro D, McGee J, Schechter R, Vugia DJ. Wound botulism in California, 1951–1998: recent epidemic in heroin injectors. Clin Infect Dis 2000;31:1018–24. 10.1086/318134 11049786

[3] Peak CM, Rosen H, Kamali A, Wound botulism outbreak among persons who use black tar heroin—San Diego County, California, 2017–2018. MMWR Morb Mortal Wkly Rep 2019;67:1415–8. 10.15585/mmwr.mm675152a3 30605447 PMC6334826

[4] Offerman SR, Schaefer M, Thundiyil JG, Cook MD, Holmes JF. Wound botulism in injection drug users: time to antitoxin correlates with intensive care unit length of stay. West J Emerg Med 2009;10:251–6. 20046244 PMC2791728

[5] Sobel SJ. Botulism. Clin Infect Dis 2005;41:1167–73. 10.1086/444507 16163636

